# VPS34 in Autophagy, Cancer, and Cancer Therapy

**DOI:** 10.3390/cells15070636

**Published:** 2026-04-01

**Authors:** Elisabetta Bartolini, Bassam Janji, Ruize Gao

**Affiliations:** 1 Tumor Immunotherapy and Microenvironment (TIME) Group, Department of Cancer Research, Luxembourg Institute of Health, 6A, Rue Nicolas-Ernest Barblé, L-1210 Luxembourg, Luxembourg; elisabetta.bartolini@lih.lu; 2 Faculty of Science, Technology and Medicine, University of Luxembourg, L-4365 Esch-sur-Alzette, Luxembourg

**Keywords:** VPS34, autophagy, immunity, cancer immunity, cancer therapy, cancer immunotherapy

## Abstract

**Highlights:**

**What are the main findings?**

- VPS34, the only class-III PI3K, is a central regulator of autophagy that controls autophagosome formation, cellular stress adaptation, and membrane trafficking through PI3P generation.
- Emerging evidence indicates that VPS34 also regulates tumor immunity and the tumor microenvironment by modulating pathways such as cGAS–STING and STAT1, thereby influencing immune cell infiltration and tumor immunogenicity.

**What are the implications of the main findings?**

- Pharmacological inhibition of VPS34 can remodel the tumor immune microenvironment and convert immunologically “cold” tumors into “hot” tumors by enhancing interferon signaling and immune cell recruitment.
- VPS34 inhibitors represent promising therapeutic agents that may synergize with immune checkpoint blockade and other anticancer therapies, highlighting their potential for future translational and clinical applications.

**Abstract:**

Autophagy is a fundamental lysosome-dependent degradation process that maintains cellular homeostasis in response to stress. VSP34 (Vacuolar Protein Sorting 34, PIK3C3) is the only class-III phosphatidylinositol 3-kinase and generates phosphatidylinositol 3-phosphate (PI3P) for auto-phagosome nucleation and maturation. Thus, it provides a critical adaptive survival pathway for cells that are experiencing metabolic stress. The VPS34–autophagy axis plays dual roles in cancer, which depend on the context: it can restrain early tumorigenesis, but in established tumors, it can promote survival in conditions of hypoxia, nutrient deprivation, and therapeutic pressure. Moreover, VPS34 shapes the tumor microenvironment (TME) through its influence on both immune and cancer cells by modulating autophagy, cGAS-STING (cyclic GMP-AMP synthase Stimulator of Interferon Genes), and STAT1 pathways. VPS34 inhibition has been reported to induce an interferon response that increases CD8^+^ T and natural killer (NK) cell infiltration and converts cold tumors into hot ones. This behavior suggests that combining VPS34 inhibitors with cancer immunotherapies could be beneficial. In this review, we summarize the molecular functions and regulations of VPS34 in autophagy and discuss recent advances linking VPS34 to tumor and cancer immunotherapy.

## 1. Introduction

Phosphoinositide 3-kinases (PI3Ks) are part of a family of lipid kinases that play important roles in cell metabolism, signaling, and other essential cellular functions in all eukaryotic cells. These enzymes include three different classes (I, II, and III) based on their structures and functions [1]. Class I consists of a catalytic and a regulatory subunit and includes four isoforms: PI3Kα, PI3Kβ, PI3Kδ, and PI3Kγ. Class II comprises monomeric proteins [2] that include three isoforms: PI3K-C2α, PI3K-C2β, and PI3K-C2γ. Only one class-III PI3K has been discovered thus far: VPS34, which is encoded by the *Pik3c3* gene in mice and *PIK3C3* gene in humans [3,4].

The VPS34 gene was first discovered through yeast genetic studies in 1990 by Herm and Emr, and VPS34 was later recognized as the ancestral form of PI3 kinase, although it continues to be classified as “class III” [5,6]. Unlike classes I and II, which generate multiple phosphoinositide products, VPS34 specifically phosphorylates the third position of the inositol ring of phosphatidylinositol (Ptdins). As a result, it produces phosphatidylinositol-3-phosphate (PtdIns3P) [7,8], which is an essential lipid for orchestrating various cellular processes, such as autophagy, membrane dynamics, and endocytic trafficking [9,10].

Through these processes, VPS34 integrates stress and nutrient signals that sustain both tumor-cell metabolism and intracellular quality control. In addition to the intrinsic functions of VPS34 in tumors, it profoundly shapes the tumor microenvironment (TME) and immune response. VPS34-mediated autophagy and endosomal trafficking in tumor cells modulate different cellular pathways, such as cGAS-STING and STAT1 signaling, which results in the activation of interferon response [11,12,13]. VPS34 also regulates cellular metabolic fitness, activation, and survival in different immune cells, such as T cells, natural killer (NK) cells, and dendritic cells (DCs) [14].

These roles allow VPS34 inhibitors to reshape the TME by modulating immune-cell infiltration and the interferon response, and they may potentially contribute to the conversion of immunologically “cold” tumors into “hot” ones. Preclinical studies have shown that targeting VPS34 pharmacologically improves tumor immunogenicity and produces synergistic effects with immune checkpoint inhibitors, which makes it a highly promising immune-metabolic target [11,14,15]. In this review, we summarize the essential functions of VPS34 in autophagy, its diverse roles in tumor progression and TME regulation, and the strategic potential of VPS34 inhibitors in increasing the efficacy of cancer immunotherapy.

## 2. Structure of VPS34 and Two Complexes

### 2.1. Molecular and Structural Features of VPS34

VPS34 is a 100-kDa protein, and its architecture comprises three domains: a C-terminal catalytic domain, which is responsible for the conversion of PtdIns into PI3P; a helical domain; and a N-terminal C2 domain. The latter domain is centrally located between the two VPS34 complexes and acts as a crucial structural and protein interaction site [16,17]. The C-terminal kinase domain is the catalytic engine of VPS34, and its function is to carry out the phosphorylation of phosphatidylinositol (PI) to produce PI3P. The kinase domain contains the ATP-binding pocket and three loops. The pocket is present between two lobes: the N-lobe and the C-lobe, which are connected by a hinge region. This pocket is smaller than the one in the other PI3Ks, which is why this feature has been exploited to design selective VPS34 inhibitors [18].

The loops of the kinase domain consist of a P-loop, an activation loop, and a catalytic loop. The P-loop binds to and phosphates ATP at the N-lobe, and the activation loop interacts with the substrate PtdIns in the C-lobe. The conserved DFG motif (aspartate-phenylalanine-glycine motif) is at the beginning of the activation loop and is critical for phosphate transfer through metal ion binding. In the catalytic loop, gamma phosphates of ATP are transferred through a conserved distal histidine regidine (DHR) motif to substrate PtdIns [19,20].

**Figure 1:**
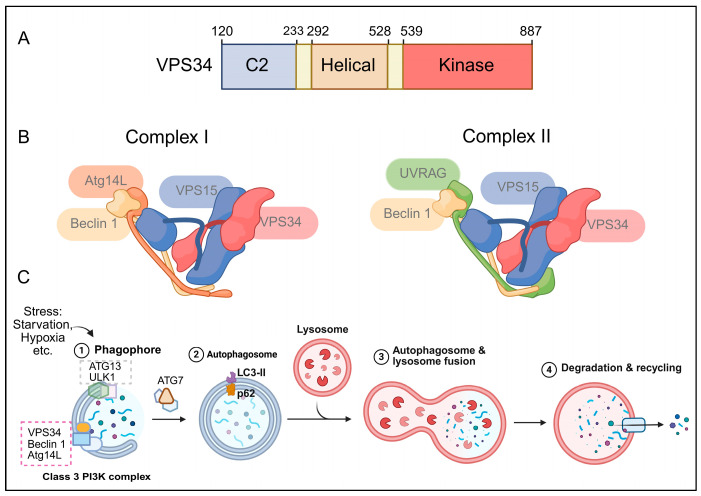
Structure of VPS34, complexes I and II, and role of VPS34 in autophagy. (**A**) Structure of VPS34: VPS34 contains a C2 domain, a helical domain, and a C-terminal kinase domain. The C2 domain mediates membrane association, the helical domain contributes to protein–protein interactions and structural stability, and the kinase domain catalyzes the conversion of phosphatidylinositol (PI) into phosphatidylinositol-3-phosphate (PI3P) [16,17,18,19,20]. (**B**) Two VPS34 complexes: VPS34 forms two major complexes with distinct functions. VPS34 complex I consists of VPS34, VPS15, Beclin 1, and Atg14L and primarily regulates autophagy initiation. VPS34 complex II consists of VPS34, VPS15, Beclin 1, and UVRAG and is mainly involved in endosomal trafficking, autophagosome maturation, and lysosomal fusion [21,22,23,24,25,26]. (**C**) The role of VPS34 in regulation of autophagy: VPS34 complex I generates PI3P at the phagophore assembly site and promotes phagophore formation. This process facilitates the recruitment of LC3 and adaptor proteins such as p62, which enables autophagosome formation. The mature autophagosome subsequently fuses with lysosomes, leading to cargo degradation and recycling [27,28,29,30]. Created in BioRender. Janji, B. (2026) https://biorender.com/ovau4r3.

The helical domain is located in the center of VPS34 and serves as a link between the C2 domain and the catalytic kinase domain. It helps to form the main structure of the “catalytic arm” in the VPS34 complex [21]. The N-terminal C2 domain is the structural hub for the complex and recruits regulatory subunits such as VPS15, Beclin 1, and Atg14L/UVRAG to form the complete complex. It contains a helical insertion C2HH (C2 Helical Hairpin), which serves as the binding site for regulatory small GTPases like Rab5 and Rab1 and directs the complex to membranes [22] (Figure 1A).

### 2.2. VPS34 Complexes I and II

VPS34 rarely functions alone and instead forms two different and mutually exclusive heterotetrameric complexes, complex I and complex II, which regulate two different functions: autophagy and endocytosis. Both complexes have a conserved V-shape structure that is evolutionarily preserved from yeast to humans [21]. Their core components include the catalytic subunit VPS34, the serine/threonine protein kinase VPS15 (also known as p150 or PIK3R4), and an accessory subunit, Beclin 1 (the mammalian counterpart of Atg6/Vps30) [22].

Beclin 1 is a ~60-kDa protein that is encoded by the *BECN1* gene in humans and *Atg1* in yeasts. It is crucial for controlling autophagy and consists of a B-cell lymphoma 2 (Bcl.2)-homology-3 (BH3) motif, a helical domain, a coiled-coil domain (CCD), an evolutionarily conserved domain (ECD), and a β/α-repeated autophagy-related domain (BARA) [23,24]. Beclin 1 binds to VPS34-VPS15 through the ECDs and CCDs and forms a Beclin 1-VPS34-VPS15 core heterotrimer. This heterotrimer interacts with Atg14L (Atg14 in yeasts) and forms Beclin 1-VPS34-VPS15-Atg14L complex I, which increases VPS34 lipid kinase activity and promotes the synthesis of PI3P and autophagy initiation [25].

It can also bind with UVRAG (UV irradiation resistance-associated gene product; Vps38 in yeasts) and form Beclin 1-VPS34-VPS15-UVRAG complex II (endosomal trafficking specific), which mediates endocytosis [26]. The structural difference between these two complexes determines their distinct cellular localizations. Atg14L directs complex I to sites of phagophore formation and ER compartments under conditions of starvation. UVRAG directs complex II to Rab9 and Rab5 positive endosomes and has a role in endocytic trafficking and autophagosome-lysosome fusion [21,22] (Figure 1B).

## 3. Canonical Functions of VPS34 in Autophagy

### 3.1. Role of VPS34 in Macroautophagy

Autophagy (particularly macroautophagy) begins with the formation of a de novo double-membraned phagophore (also called an isolation membrane). This structure expands within the cytoplasm and seals in a double membrane vesicle known as an autophagosome, which sequesters cellular components such as damaged organelles, proteins, and other materials [31]. These autophagosomes fuse with lysosomes (in mammals) or vacuoles (in yeast cells), degrade the material and recycle the cargo [32,33]. VPS34 has a central role in the regulation of autophagy and is indispensable for both its initiation and progression [34].

VPS34 complex I has a key function in the early stages of phagophore formation (initiation and nucleation), where it produces PtdIns3P on specialized membrane domains known as phagophore assembly sites (PASs) in yeast. In mammals, it arises from different locations, such as the omegasome, which is also located in the endoplasmic reticulum (ER) [28]. This process recruits downstream PtdIns3P effector proteins, including WD-repeat protein, which interacts with phosphoinositides (WIPIs), and double-FYVE containing protein 1 (DFCP1), which is important for autophagosome maturation [29].

WIPIs include four proteins (WIPI 1–4), while yeasts have three (Atg18, Atg21, and Hsv2). WIPI2b is important for promoting the lipidation of LC3 (microtubule-associated protein 1A/1B-light chain 3) and starvation-induced autophagy, which mainly occurs through the recruitment of ATG12-ATG5-ATG16L1. Findings suggest that upon sensing increased PtdIns3P levels, WIPI2b recruits the LC3 conjugation machinery to facilitate the conjugation of LC3 to the membrane of the forming autophagosome [21,35]. The lipidated form of LC3 (also called LC3-II) is attached to the membrane and fulfills the needs of cargo receptors such as p62/SQSTM1 to bind ubiquitinated substrates [30]. Some studies suggest that LC3-II, along with other ATG proteins, is involved in the contribution of the membrane curvature and closure during the last steps of autophagosome formation [36].

The mature autophagosome fuses with lysosomes and forms the autophago-lysosome through VPS34 complex II. At this stage, LC3-I located on the *inner* membrane is degraded together with the cargo, while LC3-II located on the *outer* membrane is delipidated and recycled to its soluble form, LC3-I. Therefore, LC3 (LC3-I/LC3-II) and the cargo p62 are widely used as markers to monitor autophagy flux [37] (Figure 1C).

### 3.2. Role of VPS34 in Selective Autophagy

In addition to the regulation on macroautophagy, VPS34 also plays a key role in regulating different types of selective autophagy. Selective autophagy is a precise intracellular degradation process that specifically degrades organelles, invading pathogens, and proteins aggregates for lysosomal breakdown. These processes include mitophagy (damaged mitochondria), xenophagy (pathogens), ER-phagy, ribophagy (ribosomes), and pexophagy (peroxisomes) [38,39].

In selective autophagy, VPS34 also functions as a localized signaling hub that converts PI into PI3P and recruits downstream effectors like WIPI2 and DFCP1. This process initiates autophagosome membrane (phagophore) nucleation around specific substrates. Specific receptors (or selective autophagy receptors (SARs)) are used to bridge the VPS34-dependent phagophore to enable autophagosome maturation and maintain cellular homeostasis [40,41]. In the context of mitophagy, upon mitochondrial depolarization, PINK1 accumulates on the outer mitochondrial membrane, phosphorylates, and activates Parkin, which leads to the accumulation of phospho-ubiquitin chains on multiple mitochondrial membrane proteins. These phosphorylated ubiquitin signals are recognized by receptors such as OPTN and NDP52, which link damaged mitochondria to the VPS34-dependent phagophore [42].

Xenophagy is another specialized form of selective autophagy that degrades invading pathogens like bacteria, viruses, and parasites. Pathogens are often ubiquitized and recognized by galectins, which are bound by receptors like p62 and NDP52. This process leads to recruitment of the VPS34-Atg14L complex for maturation of the autophagosome [43]. ER-phagy is the selective degradation of damaged or excess fragments of ER via lysosomes. Under stress conditions, ER-resident receptors such as FAM134B, SEC62, and RTN3 mediate the recognition and sequestration of specific ER domains. These receptors coordinate with the autophagy-initiation machinery and facilitate localized activation of the VPS34 complex in ER subdomains. VPS34-dependent PI3P production promotes phagophore formation and initiates the sequestration of ER fragments [44].

VPS34 is also essential for other selective autophagy processes like ribophagy and pexophagy, in which it generates the PI3P signal required for phagophore nucleation at organelle-specific sites. In ribophagy, the selective receptor NUFIP1 (or Heh1 in yeast) mediates the recognition and sequestration of ribosomes, whereas in pexophagy, NBR1 (or Atg30/Atg36 in yeast) targets peroxisomes for degradation. Cargo specificity is dictated by these distinct receptors, and VPS34 activity provides an indispensable membrane platform that enables receptor-guided autophagosome formation [45,46].

### 3.3. Regulation of VPS34 Complexes and VPS34 Activity

#### 3.3.1. Regulation of VPS34 Complexes I and II

VPS34 complex I is tightly regulated during autophagy in response to nutrient stress. This complex is specifically dedicated to autophagy initiation, and its activity is mainly regulated by the Unc-51-like kinase 1 (ULK1) complex through phosphorylation of key components such as Beclin 1 and Atg14L [47]. ULK1 is a serine/threonine kinase that is localized to organelles such as the ER, lysosomes, and mitochondria. ULK1 can form a stable complex with ATG13 and FIP200 regardless of nutrient conditions, which leads to altered activity of VPS34 complex I [48].

The activity of ULK1 is mainly modulated by two upstream regulators: the mammalian target of rapamycin complex 1 (mTORC1) and AMP-activated protein kinase (AMPK) [49]. mTORC1 is a key protein complex that monitors cellular energy and nutrient status, including ATP and amino acid availability, to coordinate cell growth, metabolism, and catabolic processes such as autophagy. Under nutrient-rich conditions, mTORC1 is active and phosphorylates ULK1 and ATG13, which suppresses the kinase activity of the ULK1 complex and inhibits autophagy by blocking VPS34 complex I [50,51].

Conversely, under nutrient deprivation, AMPK (the central cellular energy sensing kinase) is activated and inhibits mTORC1 activity. As a result, mTOR is dissociated from ULK1, which allows the ULK1 complex to phosphorylate ATG13 and FIP200 and triggers autophagy initiation [49,52,53,54].

In addition to regulation by the AMPK–mTORC1–ULK1 axis, VPS34 complex I activity is also modulated by competitive interactions with components of VPS34 complex II. UVRAG can compete with Atg14L for binding to the CCD of Beclin 1, which influences the function of VPS34 complex I and fine-tunes the balance between autophagy initiation, autophagosome maturation, and endocytic trafficking [25]. Moreover, Atg14L can also increase the kinase activity of VPS34 and promote autophagy initiation, but this effect appears to depend on the overexpression of Beclin 1 [55].

The activity of VPS34 complex II is mainly regulated by Rubicon (Run domain Beclin 1 interacting and cysteine-rich containing protein). Rubicon is primarily known as a negative regulator of canonical autophagy and acts as a “brake” that slows down the degradation of damaged cellular components. Mechanistically, Rubicon directly associates with UVRAG through the CCD and RUN domains within the VPS34 complex II and forms a pentameric Rubicon-UVRAG-Beclin1-VPS34-VPS15 complex. This complex blocks the activity of complex II and hinders the maturation of autophagosomes and endosomes [56,57]. Moreover, with the phosphorylation of UVRAG by mTORC1, the binding of UVRAG with Rubicon is amplified through the RUN domain, which prevents autophagosome maturation and lysosome fusion [58,59].

#### 3.3.2. Post-Translational Modifications of VPS34

Beyond interactions with various regulatory proteins and the AMPK-mTORC1 axis, VPS34 is also modulated by different stresses via specific post-translational modifications (PTMs). PTMs are protein alterations that occur after translation, which are essential for maintaining stable and regulated autophagy machinery. Under conditions of cellular stress, AMPK phosphorylates VPS34 at Thr163 and Ser165, which leads to inhibition of non-autophagic VPS34 complexes. This regulation helps to preserve cellular energy homeostasis during starvation. Under cellular stress conditions, the phosphorylation of VPS34 at Thr163 and Ser165 from AMPK inhibits non-autophagy VPS34 complexes to preserve cellular energy during starvation [60]. In contrast, autophagy-promoting VPS34 complexes are regulated by the phosphorylation of Beclin 1 at S15 (Ser14 in mice) from ULK1 and at S90/S93 (Ser91 and Ser94 in mice) from AMPK, which activate VPS34 complexes I and II, respectively [22,51,60].

VPS34 is also negatively regulated by cyclin-dependent kinases. CDK1 phosphorylates VPS34 at Thr159, which disrupts its interaction with Beclin 1. Similarly, CDK5 phosphorylates VPS34 at Thr668 within the catalytic domain. Both modifications inhibit VPS34 kinase activity [8]. Under nutrient deprivation, ULK1 phosphorylates lactate dehydrogenase A (LDHA) at Ser196, which increases intracellular lactate levels. This metabolic shift triggers VPS34 lactylation at Lys356 and Lys781, which is mediated by KAT5/TIP60. Thus, the recruitment of VPS34 to Beclin 1–Atg14L and UVRAG complexes is increased, which increases lipid kinase activity and drives the autophagy machinery forward [61]. These processes highlight the mechanisms of how VPS34 is highly regulated by the cellular metabolic state through different post-translational modifications.

#### 3.3.3. HIF-1α-Dependent Regulation of VPS34 by Hypoxia

Hypoxia is a major metabolic stress condition that greatly affects the autophagy machinery. Hypoxia involves reduced oxygen availability and is commonly found in solid tumors. Up to 90% of tumors present hypoxic regions, which are a hallmark of cancer [62]. Hypoxia is correlated with worse prognoses and the activation of hypoxia-inducible factors (HIFs), which are important transcription factors [63]. HIF-1 is a heterodimeric transcription factor with a central role in the cellular response to low oxygen levels [64].

Under hypoxic conditions, the stabilization of HIF-1α triggers the transcriptional upregulation of autophagy receptors BNIP3 and BNIP3L. Due to its better binding affinity, BNIP3 competitively displaces Beclin 1 from its inhibitory sequestration by BCL-2. This relocation makes Beclin 1 available to assemble with and activate the VPS34 complex, which initiates autophagy in response to low oxygen availability [65]. Thus, VPS34 functions as a critical downstream effector of the hypoxia signaling pathway, where its production of PI3P is indispensable for both autophagosome formation and maturation. Stabilization of HIF-1α by hypoxia also increases glycolytic flux and lactate production, which contributes to VPS34 activation [66]. These mechanisms establish VPS34 as a key downstream effector of the HIF-1α-dependent hypoxic mechanism in autophagy regulation [67].

## 4. Non-Autophagic Functions of VPS34

### 4.1. Role of VPS34 in Endosomal Trafficking and Vesicular Transport

VPS34 also plays an important role in endosomal trafficking and vesicular transport, particularly in the regulation of Rab5 activity and early endosome function. Endosomes are organelles that sort, process, and transport intracellular materials through endocytic pathways [68]. The Rab family of GTPase is important for membrane trafficking [69] and can be found in a GTP-bound active state or a GDP-bound inactive state. Rab5 and Rab7 are essential for early and late endosome trafficking, respectively, as well as the recruitment of effector proteins (particularly VPS34). The recruitment of VPS34 activates the complex through the binding of the VPS34 C2 domain to the VPS15 WD40 domain [21,70], which produces PI3P. PI3P binds to FYVE and PX domain-containing proteins and triggers endosome maturation and trafficking. Simultaneously, VPS34 also inhibits Rab5 and Rab7 through a negative feedback loop. This process recruits Rab GTPase-activating proteins (GAPs) such as TBC1D2/Armus and converts them to their inactive form [71].

### 4.2. Role of VPS34 in Phagocytosis, Lysosomal Biology, and Nutrient Sensing

Phagocytosis is a form of endocytosis that is based on the engulfment and elimination of pathogens and other particles. VPS34 has been demonstrated to regulate the formation of phagosomes and fusion with lysosomes through the production of PI3P. In the absence of VPS34, phagocytosis is impaired, which leads to the accumulation of lysosomes and a failure of phagosomes to fuse properly with these degradative compartments [72].

Moreover, VPS34 is also critical for lysosomes through PI3P production. Under nutrient-rich conditions, there is a positive loop between mTORC1 and VPS34 activation, with is followed by lysosomal PI3P production. This loop keeps the lysosomes peripheral, motile, and less degradative. When mTORC1 is inhibited, during starvation, this loop is interrupted, and the lysosomes appears larger, static, and more degradative [73].

### 4.3. Role of VPS34 in Exocytosis and Secretion

Once VPS34 activates the autophagy pathway, through the production of PI3P and following production of autophagosomes, it acts in different directions. The first direction is degradative autophagy with the fusion of autophagosomes with lysosomes and successive autophagolysosome production. In addition, there is a specific form of autophagy called secretory autophagy, where autophagosomes (containing cargo) fuse with the plasma membrane through a process called exocytosis [74]. Before plasma membrane extrusion, autophagosomes may fuse with late endosomes or multivescicular bodies (MVBs) and produce amphisomes. These organelles fuse with either lysosomes (in degradative autophagy) or plasma membrane (in secretory autophagy) [75].

Thus, secretory autophagy relies on the core VPS34–Beclin 1 machinery while redirecting downstream trafficking. This type of autophagy has recently emerged as a contributor of not only proteins, organelles, and microbes exocytosis, but also cytokines such as IL-1β, IL-6, IL-18, and TNF-α [76,77]. These insights provide new cell mechanisms of substance secretion that correlate with regulation of the tumor environment and autophagy [78].

## 5. Role of VPS34 in Cancer

As the master regulator of autophagy and endosome trafficking, VPS34 plays important roles in tumorigenesis by modulating the metabolic survival and immune evasion of tumors [79]. The roles of VPS34 in cancer cells are defined by its dual functions. Depletion or impairment of VPS34 disrupts the process of initiating autophagosome formation and endocytic trafficking to prevent cellular dysfunction, which leads to tumor initiation and progression [79,80]. However, the role of VPS34 is highly context-dependent and shifts to an opposite role based on the tumor stage and the microenvironment.

### 5.1. VPS34 as a Tumor Suppressor

VPS34 typically functions primarily to prevent cancer initiation at the early stage of transformation by driving basal autophagy to maintain cellular homeostasis and maintain genomic stability [81]. In some types of cancer, such as hepatocellular carcinoma (HCC), it has been identified as an invasion suppressor. The suppression results from the reduction in tumor metastasis by promoting lysosomal accumulation and reducing the recycling of cell-surface receptors [80].

### 5.2. VPS34 as a Tumor Promoter

In established tumors, VPS34 can paradoxically support cancer progression by promoting tumor-cell survival during metabolic stress, which supports oncogenic signaling and facilitates TME adaptation [82,83]. Interestingly, Ramos-Delgado et al. recently demonstrated an atypical role of VPS34 in cell plasticity differentiation and cancer initiation. Through deletion of VPS34 on pancreatic exocrine cells, with increased lysosomal degradation of pro-inflammatory REG3A, the newly differentiated cell state was less sensitive to cancer promotion by oncogenic KRAS in pancreatic cancer. This behavior indicates a potential opposite role of VPS34 in cancer initiation [84].

### 5.3. VPS34’S Role in Cancer-Cell Metabolism, Survival, and Metastasis

Cancer cells frequently occur in conditions of lacking nutrients and metabolic volatility. VPS34 supports cancer-cell metabolism primarily by regulating nutrient scavenging and maintaining proteostasis when the cells are under stress. VPS34-mediated autophagy provides a continuous supply of amino acids, fatty acids, and nucleotides and maintains mitochondrial TCA cycle flux and ATP production even with an external nutrient shortage [6].

VPS34-driven autophagy is a canonical adaptive response for cancer cells to survive different stresses, such as amino acid/glucose limitation, stress from therapy, and hypoxia. During chemotherapy or radiotherapy, VPS34 helps to clear proteotoxic aggregates and contributes to mitophagy, which prevents the activation of apoptotic pathways and increases resistance to therapy [42]. VPS34 also facilitates tumor metastasis by modulating the initiation of autophagy, mitochondrial homeostasis, and metabolism. VPS34 inhibits hepatocellular carcinoma invasion by regulating endosome-lysosome trafficking [80], and death-effector domain-containing DNA-binding protein (DEDD) could interact with and stabilize the VPS34/Beclin 1 complex, resulting in attenuation of epithelial–mesenchymal transition (EMT) in human breast-cancer cells [82].

Liu et al. demonstrated the role of VPS34 in promoting liver cancer stem cells (CSCs) and facilitating tumor progression. Mechanistically, VPS34 inhibition can deactivate SGK3 (a CSC promoter) and increase AMPK activation, which leads to suppression of liver CSCs [85]. Nevertheless, in another study, VPS34 was reported to promote EMT markers like SNAIL and Vimentin through stimulation of p62 phosphorylation in breast-cancer cells [86]. Islam et al. recently reported that targeting VPS34-mTORC1 signaling in dormancy-prone breast-cancer cells blunts the initiation of metastasis in a mouse model [83]. Thus, the role of VPS34 in the regulation of metastasis is largely unknown and needs further investigation.

### 5.4. Genetic Depletion in Mouse Models and Clinical and Prognostic Relevance in Human Cancers

It is well known that complete germline deletion of *Pik3c3* is embryonically lethal, whereas *Pik3c3*^+/−^ heterozygous mice are viable and fertile [87] and have not been reported to develop spontaneous tumors. Instead of tumorigenesis, conditional depletion of *Pik3c3* in different tissues has been shown to induce other diseases, such as rapid neurodegeneration [88] and Fanconi-like syndrome [89]. Mouse models targeting VPS34-related autophagy genes have demonstrated tumor-suppressive functions of autophagy in the early stages of tumorigenesis.

Beclin 1 is a core component that coordinates the assembly and activation of the VPS34 complex. Yue et al. reported that while homozygous deletion *Becn1*^−/−^ is embryonically lethal, *Becn1*^+/−^ heterozygous mice can survive, develop a higher incidence of spontaneous tumors, and develop various malignancies, such as lymphomas, lung adenocarcinomas, and hepatocellular carcinomas [90]. Liver-specific deletion of downstream proteins of VPS34 (Atg5 or Atg7) leads to benign liver adenomas [91] and accelerates tumorigenesis once hydrides with other oncogenic drivers [92].

**Figure 2:**
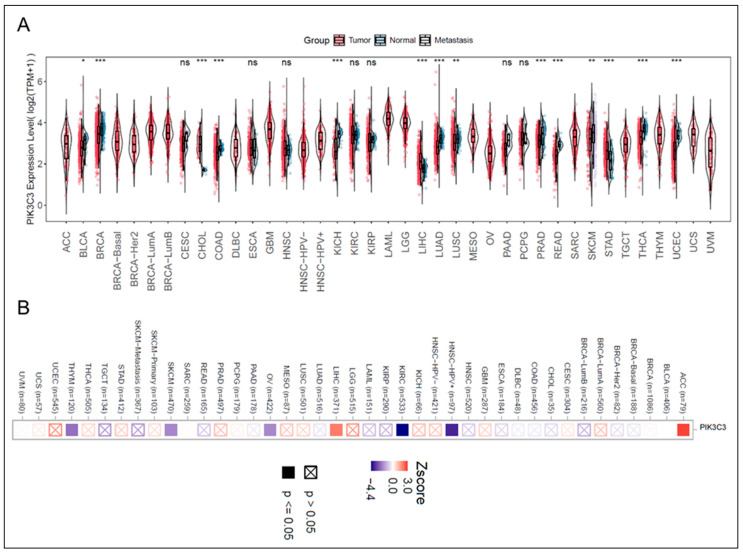
Pan-cancer mRNA expression and prognostic significance of VPS34 (PIK3C3). (**A**) mRNA expression profile of VPS34 across various types of cancer and adjacent normal tissues based on TCGA datasets. Statistical significance (* *p* < 0.05, ** *p* < 0.01, *** *p* < 0.001, ns: non-significant). Analysis using the TIMER 3 web server. (**B**) Heatmap indicates the correlation between VPS34 mRNA expression and patient survival (*Z*-score). Red indicates high expression is associated with higher risk, while blue signifies low expression as a risk factor. Crossed boxes indicate statistically significant associations (*p* < 0.05). Analysis using the TIMER 3 web server was based on TCGA datasets.

To date, no study has clearly combined canonical cancer-specific genetically engineered mouse models (GEMMs) with *Pik3c3* deletion. However, genetic depletion of *Pik3c3* can reduce tumor growth in syngeneic murine models of various cancers, like melanoma and colorectal cancer [14]. While *PIK3C3* is rarely deleted or mutated in human cancers, the expression of VPS34 depends on the context in different types of cancer. Transcriptomic analysis based on publicly available data from The Cancer Genome Atlas was analyzed using the TIMER 3.0 web server, which demonstrated that VPS34 has a relatively low mRNA expressions in most types of cancer. However, there is significantly higher expression in cholangiocarcinoma (CHOL), liver hepatocellular carcinoma (LIHC), and stomach adenocarcinoma (STAD) compared with normal tissues [93,94] (Figure 2A).

Zhu et al. investigated 60 tumor samples from patients with gastric cancer (GC) and individuals without cancer by IHC staining and found significantly lower VPS34 expression in those with GC (positive VPS34 expression: 23.3% in tumor tissues and 66.7% in adjacent tissues) [95]. Marzia et al. also reported that in patients with breast cancer, high VPS34 correlates with worse overall survival [96]. Survival probability analysis of patients with TCGA cancer using the TIMER 3 web server also indicated increased risk of patient mortality with higher VPS34 expression in some cancers like liver cancer and adenoid cystic carcinoma (ACC) [93,94] (Figure 2B). In summary, VPS34 exhibits distinct characteristics in different types of cancer and patients and needs further study to assess the possibility of targeting it to treat different cancers.

## 6. VPS34/Autophagy in Immunity and Cancer Immunotherapy

### 6.1. Role of VPS34/Autophagy in the Regulation of Innate Immunity

#### 6.1.1. VPS34/Autophagy and cGAS-STING Pathway

Innate immunity is the body’s first line of defense against invading pathogens, and the cGAS-STING pathway is a critical innate immune mechanism that detects the cytosolic DNA, viral or bacterial infection, or cellular damage. Detection triggers an immune response and the production of type I interferons (IFNs) and primarily IFN-β [97]. Autophagy and endolysosomal trafficking are functionally interconnected with cytosolic DNA sensing. As the key component that mediates autophagy, Beclin-1 has been shown to interact with cGAS. Once cGAS binds to small cytosolic dsDNA (<45 bp), it leads to the autophagic degradation of cytosolic dsDNA and reduces STING activation [98]. It has also been reported that ubiquitinated cGAS can be captured by p62 for lysosomal degradation [99].

STING is also linked to autophagy, and Tuozhi et al. recently reported that it can induce non-canonical autophagy to regulate endolysosomal homeostasis, which revealed mutual regulatory relationships between STING and autophagy [100]. Autophagy can degrade STING through different mechanisms, such as endosomal sorting complexes required for transport (ESCRT)-driven microautophagy [101]. In another mechanism, TBK1-mediated phosphorylation of p62 increases their ubiquitination level of STING and facilitates its interaction with p62, which leads to STING degradation with p62-mediated selective autophagy [102]. The interplay between cGAS-STING and autophagy has been recognized to regulate cancer progression and anti-tumor immunity in different manners. Blockading trafficking-mediated STING degradation with the autophagy inhibitor bafilomycin A1 could specifically increase STING signaling and the anti-tumor response [103].

**Figure 3:**
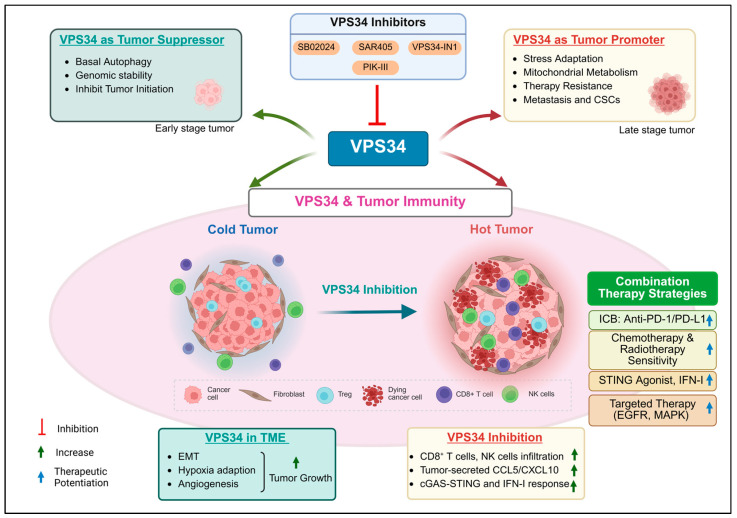
Integrated roles of VPS34 in cancer and cancer immunotherapy. VPS34 (PIK3C3) regulates autophagy, cellular metabolism, and immune signaling in cancer. In early tumorigenesis, VPS34-mediated basal autophagy maintains genomic stability and restrains tumor initiation. In established tumors, VPS34 supports tumor-cell survival under metabolic stress and promotes EMT, cancer stemness, and therapeutic resistance. Within the TME, VPS34 shapes immune responses by regulating chemokine secretion (e.g., CCL5, CXCL10), hypoxia adaptation, and angiogenesis, as well as by controlling the function of dendritic cells, NK cells, T cells, and B cells. Pharmacological inhibition of VPS34 using selective inhibitors (SAR405, SB02024, VPS34-IN1, and PIK-III) increases cGAS–STING signaling and type-I interferon responses, which promotes immune-cell infiltration and converts “cold” tumors into “hot” tumors. Combination strategies with immune checkpoint blockade, STING agonists, or conventional therapies are proposed to improve antitumor efficacy. Created in BioRender. Janji, B. (2026) https://BioRender.com/acbjivt.

VPS34 mostly plays an important role in maintaining autophagic flux and endolysosomal trafficking, which is essential for regulating cGAS-STING translocation and degradation. Recent studies have demonstrated that both pharmacologic and genetic inhibition of VPS34 would amplify cGAS–STING signaling, which leads to a type I IFN response and increased secretion of T cell-recruiting chemokines, such as CCL5 and CXCL10. Mechanistically, phosphorylation levels of TBK1 and IRF3 are significantly increased upon blocking VPS34 by treatments with either siRNA or VPS34 inhibitors [11,12,13] (Figure 3).

#### 6.1.2. Role of VPS34 in DCs and NK Cells

VPS34 is also reported to regulate DCs and NK cells. Paulo et al. recently demonstrated that blocking VPS34 in plasmacytoid dendritic cells (pDCs) with a small inhibitor such as VPS34-IN1 triggers the activation of the STING and significantly enhances pDCs’ response to the STING agonist 2′3′-cyclic guanosine monophosphate–adenosine monophosphate (2′3′-cGAMP). This leads to increased expression of type-I IFNs [104]. Moreover, VPS34 has been shown to be critical for NK cell development and senescence. A recent study generated *Pik3c3*^fl/fl^/ *CD122*^Cre/+^ mice and deleted *Pik3c3* during and after NK-cell commitment. The results revealed that VPS34-mediated vesicular transport is important for CD122 membrane trafficking during NK-cell commitment, and VPS34-mediated autophagy can delay NK cell senescence [105].

### 6.2. Role of VPS34/Autophagy in the Regulation of Adaptive Immunity

Adaptive immunity is the third line of defense against infectious and malignant diseases, especially malignant tumors [106]. Unlike innate immunity, adaptive immunity is a highly specialized system for antigen recognition and is responsible for long-lasting immune-system memory [107]. Adaptive immunity is mediated by lymphocytes (mostly B-lymphocytes and T-lymphocytes). Autophagy has been shown to play important roles in modulating several processes in adaptive immunity, such as lymphocyte metabolism, T-cell activation and differentiation, and B-cell activation and development [108].

Autophagy is essential for antigen processing and presentation, especially MHC-I in the context of DC cross-presentation to CD8^+^ T cells and MHC-II for CD4^+^ T cells. The homeostasis, activation, and differentiation of T and B lymphocytes in particular modulated by autophagy to maintain lymphocyte heath [109]. As the key component of autophagy, VPS34 is essential for autophagic processes in lymphocytes and underpins fundamental aspects of T-cell biology.

Willinger et al. generated conditional *Pik3c3* KO mice and T cells and demonstrated the essential role of *Pik3c3* for the homeostasis of naïve T cells (CD4^+^ and CD8^+^ T cells), but not T-cell development. *Pik3c3*-deficient T cells show accumulation of reactive oxygen species (ROS), which is consistent with deficient removal of damage mitochondria in a canonical autophagy manner [110]. Courreges et al. have also demonstrated the role of VPS34 in regulatory T (Treg) cells: loss of VPS34 induces a state of heightened metabolic activity that may interfere with metabolic networks that are required for maintenance or suppressive functions of Treg cells [111].

Luc et al. showed VPS34’s function in thymic epithelial cells (TECs) for CD4^+^ T selection and demonstrated that deletion of the *Pik3c3* gene in TECs causes severe defects in positive selection of the CD4 T-cell lineage, but not the CD8 T-cell lineage [112,113]. Yang et al. revealed the role of VPS34 in T-cell metabolism and function. They found that *Pik3c3*-deficient T cells exhibited impaired cellular metabolism, and *Pik3c3*-deficient CD4^+^ T cells failed to differentiate into T helper 1 cells with reduced active mitochondria upon T-cell activation. However, they observed no impact of tumor metastasis upon *Pik3c3* deletion in T cells [114,115]. The high dependence of activated T cells on VPS34 indicates a critical therapeutic tradeoff: while targeting VPS34 in tumor cells improves antitumor immunity, systemic blockade may simultaneously compromise T-cell survival.

DC homeostasis and antigen cross-presentation are tightly regulated by autophagy and VPS34-mediated autophagy in adaptive immunity. In 2017, Vrajesh et al. generated DC-specific *Pik3c3*-deficient mice and found that *Pik3c3*-deficent DCs show a partially activated phenotype, spontaneously produce cytokines, and exhibit increased activity of the classic MHC class I and class II antigen-presentation pathways. Mechanistically, VPS34 orchestrates endosomal maturation and autophagosome–endosome fusion and maintains the delicate balance between antigen degradation and effective cross-presentation. However, the animals displayed a defect in the homeostatic maintenance of splenic CD8α^+^ DCs and cross-presentations with MHC class I-restricted T cells [116,117].

Monaci et al. reported that hypoxia-induced autophagy in DCs is mediated by VPS34. Treatment with a specific VPS34 inhibitor, SAR405, abolished autophagy and affected survival and inflammatory cytokine expression in hypoxic LPS-treated DCs [118]. However, the role of VPS34 in B cells is still largely unknown. Recently, Luc et al. conditionally deleted *Pik3c3* in pro-B cells and demonstrated increased ROS levels in *Pik3c3*-deficient B cells with an anti-inflammatory cytokine profile, cell homeostasis disruption, and altered immune response capacity [119].

### 6.3. VPS34’S Role in Shaping the TME

VPS34 activity in malignant tumors and adjacent tissues shapes the TME through multiple non-mutually exclusive mechanisms. It regulates the secretome of tumor cells (notably chemokines and cytokines, e.g., CCL5 and CXCL10) to dictate immune-cell recruitment. Furthermore, it maintains tumor-cell viability under hypoxic and nutrient-deprived conditions, thereby affecting antigen accessibility. VPS34 also regulates endothelial cell migration and the processing of angiogenesis factors, which influences vascular perfusion and immune cell extravasation.

Studies using B16-F10 and CT26 syngeneic mouse models have shown that VPS34 inhibition (via either shRNA-mediated silencing or pharmacological inhibitors, such as SB02024 and SAR405) increases the infiltration of M1-like tumor-associated macrophages, NK cells, and effector T cells. Concurrently, VPS34 targeting appears to reduce the immunosuppressive activity of Tregs and myeloid-derived suppressor cells (MDSCs), thereby reprogramming the TME toward a more immunostimulatory state [14]. Moreover, VPS34 inhibition in tumors increases the cGAS-STING pathway, which leads to greater tumor control through immune-mediated mechanisms [11].

Hypoxia is the main feature of the TME, and hypoxia-induced autophagy in cancer cells supports their survival. Hypoxia-induced autophagy in endothelial cells (ECs) and blood vessels also regulate pathological angiogenesis and hypoxia adaptation and reshapes the TME [120,121]. Inhibition of VPS34 abolishes hypoxia-induced autophagy in DCs; it decreases pro-survival signaling and viability; and it increases pro-inflammatory cytokines [118]. However, there is no direct evidence demonstrating VPS34’s role in tumor angiogenesis and hypoxia adaptation (Figure 3).

## 7. Pharmacological Targeting of VPS34

### 7.1. Overview of VPS34 Inhibitors

The development of highly potent and specific VPS34 inhibitors is crucial for elucidating the role of this kinase in membrane transport and autophagy. Unlike pan-PI3K compounds, these small molecules specifically target the catalytic pocket of class III PI3K, which enables the investigation of VPS34-dependent biological mechanisms in cancer models. Several specific VPS34 inhibitors have been developed, such as SAR405 from Sanofi [122], VPS34-IN1 from the University of Dundee and AstraZeneca [123], SB02024 from Sprint Bioscience [124], and PIK-III from Novartis [125]. These inhibitors have primarily been used in models of cancer and neurodegenerative disease, where they have been shown to inhibit tumor growth and, in certain contexts, promote the clearance of protein aggregates. Targeting VPS34 with these inhibitors has already shown impressive impacts on tumor growth and chemotherapy resistance.

SAR405 is a potent, selective, and orally bioavailable VPS34 inhibitor, and by blocking the conversion of PI into PI3P, it suppresses autophagic flux and perturbs late endosomal trafficking. Concomitant inhibition of VPS34 and mTOR with SAR405 and everolimus results in synergistic anti-proliferation effect in renal tumor cells [122]. Blocking VPS34 with SAR405 in head-and-neck cancer increases the antitumor efficacy of cisplatin by modulating cancer-associated fibroblasts [126]. A similar impact has also been discovered with urothelial carcinoma cells and pleural mesothelioma cells by combining cisplatin with SAR405. This demonstrates that targeting the autophagic machinery by SAR405 might be a suitable approach to overcome cisplatin resistance in urothelial carcinoma and pleural mesothelioma [127,128]. Further studies have validated the impact of VPS34 inhibition by SAR405 in suppressing tumor growth in syngeneic tumor models [11,14].

VPS34-IN1 is a highly selective VPS34 inhibitor with minimal cross-reactivity toward class-I PI3Ks. It induces a rapid 50–60% reduction in SGK phosphorylation, which inhibits tumor growth and drug resistance [129,130]. Meunier et al. reported that VPS34 inhibition driven by VPS34-IN1 induces apoptosis of acute myeloid leukemia (AML), but not CD34^+^ hematopoietic cells. This occurs via inhibition of basal and L-asparaginase-induced autophagy, which blocks mTORC1 signaling and the FLT3-ITD-STAT5 pathway [131]. VPS34-IN1 has also shown significant impacts on breast-cancer cell proliferation and spheroid growth [96]. Wu et al. demonstrated that VPS34-IN1 significantly reduces tumor growth of ER+ breast cancer by activation of the PERK/ATF4/CHOP pathway [132], and a study on liver cancer indicated tumor-growth inhibition by VPS34-IN1 as well [85].

SB02024 is a next-generation VPS34 inhibitor with favorable pharmacokinetic properties. SB02024 has shown a significant impact on xenograft growth with MDA-MB231 and MCF-7 breast-cancer cells. Further evidence from monolayer cultures has demonstrated significant potentiated cytotoxicity of Sunitinib and eroltinib against breast-cancer cells [124]. VPS34 inhibition by SB02024 induces activation of the STAT1-IRF7 and cGAS-STING pathways, which results in robust antitumor activity in vivo in syngeneic models, where the combination of SB02024 and immune checkpoint blockade (ICB) achieved significantly enhanced tumor growth inhibition relative to monotherapy groups [11,14]. PIK-III also exhibits marked selectivity for VPS34 (reported up to ~100-fold over other PI3K isoforms) and effectively blocks autophagy [125]. Kobylarz et al. reported that the impact of VPS34 inhibition with PIK-III on RKO cancer cells impairs iron mobilization via the VPS34-RAB7A axis [133] (Figure 3).

### 7.2. Other VPS34 Inhibitors

Some potential compounds are also being developed to block autophagy and inhibit tumor growth. Liu et al. discovered a highly potent ATP-competitive PI3Kδ/VPS34 dual inhibitor named PI3KD/V-IN-01, which displayed 10 to 1500-fold higher selectivity than other PI3K isoforms and did not inhibit any other kinases in the kineme. PI3KD/V-IN-01 exhibited better inhibition against AML, CLL, and Burkitt lymphoma cell lines than other known inhibitors [129].

MPT0L145 is another VPS34 inhibitor and was initially identified as a novel FGFR inhibitor [134]. It has since been characterized as a potent VPS34 inhibitor with a *Kd* value of 0.53 nmol/L. In bladder-cancer models, MPT0L145 exhibits significant antitumor activity by inducing mitochondrial dysfunction, ROS accumulation, and DNA damage, ultimately overcoming cisplatin resistance [135]. The role of MPT0L145 in the inhibition of tumor growth and reversing targeted therapy/chemotherapy resistance has been validated with different types of cancer [136].

## 8. Combination Strategies: Synergizing VPS34 Inhibition with Other Cancer Therapies in

### 8.1. Synergizing VPS34 Inhibition with ICB

Preclinical evaluation of selective VPS34 inhibitors, such as SAR405 and SB02024, has demonstrated potent therapeutic efficacy when combined with ICB. Combining VPS34 SAR405 and SB02024 with anti-PD-1/PD-L1 therapies is an active preclinical strategy. SB02024 and SAR405 treatments for melanoma and colorectal cancers induce the infiltration of NK, CD8^+^, and CD4^+^ T effector cells and establish a T-cell-inflamed TME by modulating the STAT1-IRF7-CCL5/CXCL10 axis. Combing SAR405/SB02024 with anti-PD-1/PD-L1 also or improves the therapeutic benefit and prolongs survival in mice [14,137]. Zhang et al. demonstrated that pharmacologically targeting VPS34 resulted in increased apoptosis of neuroblastoma cells and a notable reduction in tumor growth in vivo. Notably, VPS34 inhibition significantly increased the efficacy of anti-GD2 antibodies in promoting antitumor immune responses in vivo in animal experiments [15].

### 8.2. Synergizing VPS34 Inhibition with STING Agonists

VPS34 inhibition can also amplify cGAS–STING signaling and thereby increase tumor production of Th1-type chemokines (e.g., CCL5, CXCL10). This process converts immunologically “cold” tumors into more inflamed and T cell-permissive lesions. Yu et al. reported that blocking VPS34 by SAR405 or SB02024 activates the cGAS-STING pathway and increases the type I IFN response with IFNB1, IRF1, IRF7, and IRF9 elevation in renal cancer and melanoma cells [11]. Combination therapy with SAR405/SB02024 and STING agonists leads to greater tumor control through immune-mediated mechanisms by enhancing the cGAS-STING pathway. VPS34 inhibition has shown promising impacts on remodeling the TME. However, given its role in regulating T-cell fitness, clinical translation will require sophisticated strategies such as intermittent dosing or tumor-targeted delivery to mitigate the potential deleterious effects on systemic lymphocyte fitness and essential physiological functions.

### 8.3. Synergizing VPS34 Inhibition with Other Therapies

In advanced cancers, inhibiting autophagy represents a significant clinical strategy. Blocking the cellular recycling systems that tumors rely on to counteract treatment-induced stress increases tumor sensitivity to chemotherapy, radiotherapy, and targeted therapies [138,139]. However, most preclinical studies have focused on chloroquine (CQ)/HCQ, bafilomycin A1, and other drugs instead of VPS34 inhibitors [140]. Some studies have shown an impact of different VPS34 inhibitors on improving cisplatin efficacy and overcoming chemotherapy resistance for different cancers [126,127,128]. SB02024 has also shown significant impacts of enhancing sunitinib and eroltinib’s effects on breast-cancer cells [124]. However, no study has reported on the synergized effects of VPS34 inhibition and radiotherapy on cancers. Given the pivotal role of VPS34 in regulating autophagy, blocking its effects with such therapies could benefit patients with cancer.

## 9. Conclusions and Perspectives

A major challenge of systemic VPS34 inhibition is the preservation of immune competence and particularly T-cell function. VPS34 is essential for T-cell metabolism, survival, homeostasis, and memory formation, meaning prolonged or non-selective VPS34 blockade may impair adaptive antitumor immunity. Feasible strategies to mitigate this limitation include intermittent (pulse) dosing and tumor-targeted delivery approaches. Short-term, transient VPS34 inhibition may induce intrinsic tumor inflammatory signaling and promote the recruitment and activation of immune effector cells while allowing systemic lymphocytes to fully recover. Furthermore, tumor-targeted delivery strategies such as antibody-drug conjugates, tumor-targeting nanoparticles, or other selective delivery platforms could increase VPS34-inhibitor accumulation in the TME while minimizing off-target effects in peripheral immune cells and normal tissues.

Although VPS34 inhibition has been shown to impair T cells’ metabolic adaptability and plasticity, its precise mechanism of action is not completely clear. Notably, the role of VPS34 in tumor immunity may depend on the TME and physiological state, particularly in terms of tumor-infiltrating T-cell dysfunction and the functional regulation of other immune cell populations, including tumor-associated macrophages. These context-dependent effects underscore the necessity of mechanistic studies to optimize VPS34-targeting therapeutic strategies while preserving anti-tumor immune responses.

In this context, the integration of multi-omics approaches, including transcriptomic, proteomic, and spatial analyses, will be instrumental in dissecting the complex roles of VPS34 within the tumor microenvironment, as well as in guiding patient stratification and the clinical implementation of VPS34-targeted therapies [141]. In addition, future studies should prioritize establishing the causal role of VPS34 in tumor development and progression by moving beyond correlative observations. This will require the use of well-designed functional approaches, including genetic models, temporally controlled perturbation systems, and in vivo validation strategies, as exemplified by recent studies employing rigorous experimental frameworks to dissect causality in cancer biology [142].

## Data Availability

No new data were created or analyzed in this study.
